# An International Validation of the Stigma Impact Scale With People With Dementia

**DOI:** 10.1002/gps.70123

**Published:** 2025-07-03

**Authors:** Jem Bhatt, Sara Evans‐Lacko, Katrina Scior, Rob Saunders

**Affiliations:** ^1^ UCL Unit for Stigma Research University College London London UK; ^2^ Care Policy and Evaluation, Centre London School of Economics and Political Science London UK; ^3^ Research Department of Clinical Educational and Health Psychology CORE Data Lab Centre of Outcomes Research and Effectiveness University College London London UK

**Keywords:** dementia, discrimination, psychometrics, stigma

## Abstract

**Objective:**

A robust psychometric instrument is imperative to measure the devastating impact of self‐stigma in dementia to adequately inform policy and practice. Our objective was to evaluate the psychometric properties of the Stigma Impact Scale in a global sample of people with dementia.

**Method:**

Data were analysed from the World Alzheimer Report including 710 participants in 42 countries who completed the SIS. Detailed psychometric analyses of the SIS included estimating reliability, convergent validity with the Warwick‐Edinburgh mental Well‐being Scale (WEMWBS) and the Dementia Quality of Life instrument (DQoL), the factor structure of the measure (through both exploratory and confirmatory factor analysis).

**Results:**

The SIS and its subscales had ‘good’ to ‘excellent’ internal consistency (Cronbach's Alpha: 0.883–0.943). However, convergent validity correlations were not in the predicted direction; no significant correlations were noted between the SIS and the WEMWBS and DQoL. Factor analysis suggested marginal improvements in global fit indices for the observed model compared to the theoretical model, though none met the thresholds for acceptable fit. The final proposed model had three factors: rejection and secrecy, loneliness and belonging and perceived social isolation. Most SIS items were strongly endorsed by participants.

**Conclusion:**

The SIS is the most robustly tested instrument measuring self‐stigma in dementia. The SIS has good to excellent reliability and relevance to the target population, however future work is required to improve the factor structure of the scale. Further the results of the validity testing pose a number of theoretical and empirical questions for future research.

## Summary

- Measuring self‐stigma in dementia with a robust, validated psychometric instrument would mean we could identify areas of concern for policy and practice.
- The Stigma Impact Scale (SIS), measuring self‐stigma in dementia, has been a valid and reliable measure in small samples but has never been subject to vigorous psychometric testing
- All SIS items were endorsed by approximately 20% or more of people with dementia which suggests SIS items reflect relevant and identifiable constructs that resonate with the experience of dementia
- Results of the current study confirm that SIS is a reliable measure in a global sample of people with dementia however validity and factor structure require further research efforts.

## 1 Introduction

The WHO World Health Assembly endorsed the ‘Global Action plan on the public health response to dementia 2017–2025’ which framed tackling stigma as a way of ensuring equity and access to the things people with dementia need the most to maintain a sense of autonomy and self [1]. It emphasised stigma as a barrier to social participation [1] which aligns with previous research that has noted the negative internal consequences of stigma for people with dementia ‐ this can also be referred to as ‘self‐stigma’ [2, 3, 4].

‘Self‐stigma’ refers to negative feelings and behaviours directed towards oneself as a result of a stigmatised characteristic such as a diagnosis of dementia [3]. Consequences of self‐stigma in dementia may hinder the uptake of clinical services, these include diagnostic secrecy leading to social isolation [5, 6], withdrawal and increased depression [7], delays in help‐seeking and reduced confidence [8, 9, 10] and social isolation and loneliness [11].

There is no gold standard instrument to approach the study of self‐stigma in dementia however, the Stigma Impact Scale (SIS) is the most widely used and cited tool. The SIS is based on the Multidimensional Model of Perceived Stigma which explains self‐stigma through social isolation, social rejection and internalised shame as well as insight into one's deteriorating cognitive functioning are necessary pre‐requites without which stigma cannot have an impact on one's sense of self [12]. The SIS has been used in various countries (USA, UK, Italy, the Netherlands and Poland) to understand the stigma experiences of people with dementia in samples ranging from 41 to 180 participants [13, 14, 15, 16, 17]. These studies have used the version omitting the financial insecurity sub‐scale due to lack of relevance for people with dementia following consultation with lived experience experts and poor internal consistency [18]. The most recent studies using the SIS have found the scale to excellent internal consistency for the overall scale total (Cronbach's alpha = 0.906) but a range of poor to excellent internal consistency for subscales (Cronbach's alpha = 0.614 to 0.869), as well as evidence of convergent validity in line with pre‐specified hypotheses between stigma impact and self‐esteem [16].

The underlying theoretical model of the SIS (the Multidimensional Model of Perceived Stigma) has not been subject to evaluation in a large scale global sample nor have assumptions about factor structure been investigated. The literature until now clearly points towards stigma exacerbating the negative experiences of people with dementia. It is therefore important that we test the underlying theoretical model of the SIS to see whether it is relevant for people with dementia. Further, a reliable and valid measure of self‐stigma in dementia has implications for policy, practice, research and innovation. Measuring self‐stigma in dementia with a robust, validated psychometric instrument would mean we could identify areas of concern for policy and potentially integrate the instrument into practice to understand ways in which stigma affects the lives of people with dementia. This in turn would lead to gathering data on innovative ways to reduce or lessen this stigma. Here we examine the psychometric properties of the SIS in a global sample of people with dementia assessing the reliability, validity and factor structure of the SIS. We will also investigate the extent to which items of the SIS are endorsed in a global sample to understand the relevance of the measure for people with dementia.

## 2 Materials and Methods

Data were gathered as part of a cross‐sectional survey for the World Alzheimer Report 2019 commissioned by Alzheimer Disease International (ADI), a full technical report outlining the commissioned survey, recruitment methodology and sampling can be found elsewhere (https://www.alzint.org/resource/world‐alzheimer‐report‐2019/). In this paper the focus is on people who self‐identified as having dementia. To widen access, the survey was translated into 32 different languages by ADI member organisations and staff adhereing to the WHO guidelines [19], for more information please see the full technical report.

### 2.1 Participants

People with dementia (*n* = 1237) from 42 countries responded to this survey. ADI partner organisations were used to recruit participants via webinars were ran in English and Spanish to discuss recruitment. After discussions, partner organisations recruited through online platforms, online forums, social media, the ADI website, mailing lists, national Alzheimer Associations, health and social care organisations, groups that support people with dementia, charity and faith‐based organisations and word of mouth. Written informed consent was obtained from all participants.

Qualtrics, an online survey platform, was the primary method used to collect data. To ensure representation of participants from rural areas and those without internet outreach work via healthcare and community professionals was conducted using hardcopy forms or offline data collection through the use of Mobenzi for Windows (Mobenzi Technologies, Cape Town, South Africa; see https://www.engineeringforchange.org/solutions/product/mobenzi/). This research was granted ethical approval by the London School of Economics and Political Science self‐certification process (Reference: CPEC‐LSE‐2019‐SE‐06). There was an option of completing the survey through proxy (via support by a family member, health worker or third sector workers), respondents were alerted to tick the ‘proxy’ option at the beginning of the survey if this was the case.

### 2.2 Measures

#### 2.2.1 Stigma Impact Scale

The original Stigma Impact Scale (SIS) [12] consists of 21 items. In the current study 1 item was removed (item 21 ‘changes in my appearance have affected my social life’) following stakeholder feedback about it being irrelevant and therefore a 20‐item version was used more information on stakeholder feedback can be found elsewhere (https://www.alzint.org/resource/world‐alzheimer‐report‐2019/). Each item was rated on a Likert scale from 1 (strongly disagree) to 4 (strongly agree), with additional score of 0 recorded for ‘not applicable’. Higher total scores indicated higher levels of stigma impact. The SIS structure consisted of three subscales, internalised shame, social rejection and social isolation, as per aforementioned studies the financial insecurity scale was omitted participants [13, 15, 16, 17]. Previous literature in smaller samples suggests the SIS overall has excellent internal consistency (Cronbach's alpha: 0.91) and subscales have poor to excellent (Cronbach's alpha = 0.614 to 0.869) [16].

#### 2.2.2 Additional Measures

##### 2.2.2.1 Warwick‐Edinburgh Mental Wellbeing Scale (WEMWBS)

The WEMWBS is a 14‐item measure designed to assess mental wellbeing [20] which has been robustly tested [21]. Although the WEMWBS is not dementia‐specific, several studies have used the measure with people with dementia [22]. The WEMWBS has been used to assess wellbeing in dementia [22] and performed well in a global sample of people with dementia [23]. Items are answered on a five‐point Likert scale ranging from 1 (none of the time) to 5 (all of the time). Higher scores represent greater wellbeing and scores range from 14 to 70. The WEMWBS has good internal consistency and test re‐test reliability (Cronbach's alpha: 0.94; McDonald's *ω* = 0.95) [21].

##### 2.2.2.2 Dementia Quality of Life Instrument (DQoL)

The DQoL is a dementia‐specific measure of quality of life developed for use with individuals with mild to moderate dementia [24]. Although the original scale had five subscales, only three were used (negative affect, feeling of belonging and self‐esteem) as a result of feedback stakeholder and stigma experts deeming the other subscales (positive affect/humour, and sense of aesthetics) irrelevant. Further, the SIS had previously shown associations with the negative affect, feeling of belonging and self‐esteem sub‐scales but not with positive affect/humour and sense of aesthetics [14]. Each subscale (negative affect, feeling of belonging and self‐esteem) respectively had adequate internal consistency (Cronbach's alpha: 0.89, 0.67, 0.80) and test re‐test reliability (Person's correlation coefficient: 0.64, 0.74, 0.68) [24]. Higher scores indicated greater subjective quality of life.

##### 2.2.2.3 Sociodemographic Characteristics

Data on country or territory of residence, gender, age, level of education, urbanicity and employment status were collected.

### 2.3 Data Analysis

#### 2.3.1 Missing Data and Data Preparation

Missing data patterns were evaluated using Little's test for missing at random, following established guidelines to determine appropriate handling strategies [25, 26]. For data missing completely at random (≤ 15% of item‐level responses), mean imputation was applied, whereas systematically missing data were excluded, and only complete cases were analysed. Data analysis were conducted in IBM SPSS Statistics (Version 27) and R (Version 4.3.2). In order to carry out the exploratory factor analysis (EFA; *n* = 357) and confirmatory factor analysis (CFA; *n* = 353), stratified randomisation was used ensuring similar representation from each WHO region within the two halves.

### 2.4 Psychometric Properties: Reliability and Validity

Psychometric properties of the SIS such as internal consistency (Cronbach's alpha) and convergent validity hypotheses were assessed (correlations). It was hypothesised that there would be moderate positive correlations between the SIS and the WEMWBS and the DQoL as previous research has found wellbeing and quality of life negatively associated with stigma [14, 22]. These statistical analyses were conducted on the theoretical model (the Multidimensional Model of Perceived Stigma).

### 2.5 Factor Analyses

EFA was used to explore the factor structure of the SIS. Eigenvalues, scree plots and factor loadings (≥ 0.3) [27] were used to assess the factor structure and submit a model for evaluation using a confirmatory factor analysis (CFA) in the second split sample. The EFA was conducted using the maximum likelihood method for extraction with oblique rotations, and components with eigenvalues over 1, in line with Kaiser's Criterion, were used to understand the factor structure.

The Lavaan Package (Version 0.6–18) in R was used to conduct a CFA on the factor structure found in the EFA. We examined the ‘model fit’ or ‘goodness of fit’ between observed factors and the underlying latent structure generated by the EFA. Model fit was evaluated using guidelines by Petscher et al. [28], this included the Chi‐square test statistic, Comparative Fit Index (CFI, > 0.90 acceptable, > 0.95 indication of good fit) and Root Mean Square Error of Approximation (RMSEA, > 0.06 and < 0.08 are considered acceptable).

#### 2.5.1 Endorsement of the SIS

Endorsement of SIS items was calculated as the percentage of participants who responded either ‘strongly agree’ or ‘agree’ to each item based on the assumption that these responses signify that the content of the items resonated with the participant in a manner that can be understood as *endorsing* that aspect of stigma impact in their lives. Endorsement was calculated for the overall sample and WHO regions.

## 3 Results

A total of 1237 participants with dementia completed the survey. However, in 527 cases, SIS items 12–20 were missing systematically and therefore excluded from the datasets, where only complete cases were used. The majority of the sample completed the study online independently (*N* = 608; 86%) while others required support (*N* = 48). The majority of participants had a formal diagnosis given to them by a neurologist (39.3%), other professionals included Geriatricians (15.5%), general practitioner (11.1%) and psychiatrist (7.7%), some participants selected ‘other’ (19.3%). Data from 710 participants in 42 countries were analysed, descriptive statistics of the sample can be found in Table 1. The majority of the sample were female, retired, educated to university level (60.6%), living in an urban area and from high‐income countries. Participants were mostly from Europe, the Americas and the Western Pacific Region.

**TABLE 1:** Descriptive characteristics of participants.

Variable	*N* (%) or mean (SD)
Sex	
Male	277 (39.00)
Female	433 (61.00)
Age	
*N* = 710, range: 24–92	64.81 (11.71)
Employment status	
Full time paid employment	101 (14.20)
Part time paid employment	26 (3.70)
Self‐employed	50 (7.00)
Unpaid/voluntary work	55 (7.70)
Unpaid carer	16 (2.30)
Retired	391 (55.10)
Student	4 (0.60)
Illness/sick‐leave	43 (6.10)
Looking for/other, unemployed	54 (7.60)
Education	
Less than primary/elementary school	5 (0. 70)
Primary/elementary school	22 (3.10)
Secondary school/high school (or equivalent)	163 (23.00)
Vocational training or apprenticeship	90 (12.70)
College/pre‐university/university	257 (36.20)
Post graduate degree completed	173 (24.40)
Area of residence	
Urban	332 (45.40)
Suburban	162 (22.80)
Semi‐rural	155 (21.80)
Rural	59 (8.30)
Stigma impact scale	
*N* = 710, Range: 78.00	42.35 (16.38)
WEMWBS total	
*N* = 681, range: 1.70	44.40 (11.28)
DEMQoL total	
*N* = 596, range:2.79	1.99 (0.30)
WEMWBS categorical	
Higher mental wellbeing ≥ 42	408 (57.46%)
Lower mental wellbeing (0–41)	266 (37.46%)
DQoL categorical	
Higher QoL (> median 2.25)	103 (14.51%)
Lower QoL (≤ median 2.25)	493 (69.43%)
WHO region	
African region	8 (1.13%)
Eastern Mediterranean region	5 (0.70%)
European region	317 (44.65%)
Region of the Americas	241 (33.94%)
South‐East Asia region	29 (4.08%)
Western Pacific region	110 (15.49%)
World bank income categories	
High‐income economies	580 (81.69%)
Upper‐middle economies	89 (12.54%)
Lower‐middle economies	41 (5.77%)

### 3.1 Reliability and Validity

The SIS and subscales (based on the original theoretical solution) had excellent internal consistency, with only minor improvements observed when two items were removed; therefore no items were removed based on the reliability statistics (see Table 2).

**TABLE 2:** Psychometric Properties of the Stigma Impact Scale and subscales.

Construct	Sub‐component	Theoretical model				EFA proposed model			
		SIS total	Social rejection	Social isolation	Internalised shame	Total	F1	F2	F3
Reliability	Internal consistency^a^	0.943	0.889	0.883	0.888	0.953	0.932	0.898	0.867
	Item if deleted	Minor increase if SIS1 (0.948) or SIS17 (0.944) were removed	Minor increase if SIS1 were removed 0.904	None	None	None	None	None	None
Convergent validity^b^	WEMWBS	0.039	0.028	0.093*	−0.012	−0.016	−0.069	0.065	0.011
	DQoL	0.026	0.022	0.001	0.039	0.008	−0.029	0.061	0.025

^a^ Cronbach's alpha.

^b^ Persons correlation coefficient.

*p < 0.05.

**p < 0.001.

The convergent validity hypotheses were not supported, the Pearson's correlation statistics are presented in Table 2. The results of the convergent validity analysis were not as predicted. A weak, significant, positive correlation was found between social isolation and WEMWBS scores, suggesting that as wellbeing increases, so does social isolation. No significant correlations were noted between SIS and DQoL scores.

### 3.2 EFA

Bartlett's test of sphericity was significant (*X* ^2^ (190) = 4443.248 *p* < 0.001.) and the Kaiser‐Meyer‐Olkin Measure of sampling adequacy (KMO = 0.946) was greater than 0.60 indicating the data were suitable for EFA. Three components with eigenvalues over 1 in line with Kaiser's criterion, explained 57.38% of the variance. Factor loadings can be found in Table 3 and reflect a three‐factor structure similar to that of the theoretical model with some alterations.

**TABLE 3:** Structure factor loadings for exploratory factor analysis of the stigma impact scale (*N* = 357).

	Factor 1	Factor 2	Factor 3
SIS8: I feel others think I am to blame for my dementia	0.916	−0.110	
SIS11: I feel a need to keep my dementia a secret	0.907		−0.192
SIS16: I feel I am at least partially to blame for my dementia	0.738	0.115	−0.106
SIS5: I feel others are concerned they could catch my dementia through contact like a handshake or eating food I prepare	0.737	−0.212	0.201
SIS7: Some family members have rejected me because of my dementia	0.693	−0.227	0.305
SIS10: I fear someone telling others about my dementia without my permission	0.685	0.197	−0.123
SIS9: I do not feel I can be open with others about my dementia	0.582	0.209	
SIS12: I feel some friends have rejected me because of my dementia	0.523	0.142	0.191
SIS19: Due to my dementia others seem to feel awkward and tense when they are around me	0.447	0.329	0.150
SIS13: I have a greater need than usual for reassurance that others care about me		0.835	
SIS14: I feel lonely more often than usual		0.818	
SIS17: I feel less competent than I did before my dementia	−0.246	0.757	
SIS15: Due to my impairment I have a sense of being unequal in my relationship with others		0.710	
SIS20: Due to my dementia I sometimes feel useless	0.128	0.581	0.118
SIS18: I encounter embarrassing situations as a result of my dementia	0.151	0.465	0.115
SIS2: Some people act as though I am less competent than usual	−0.210	0.195	0.775
SIS3: I feel I have been treated with less respect than usual by others		0.129	0.692
SIS4: I feel set apart from others who do not have dementia		0.159	0.657
SIS6: I feel others avoid me because of my dementia	0.333		0.593
SIS1: My employer/co‐workers have discriminated against me because of my dementia	0.165		0.191

Factor 1 contained 9 items (SIS: 5, 7, 8, 9, 10, 11, 12, 16, 19) and was named ‘rejection and secrecy’. Factor 2 contained six items (SIS: 13, 14, 15, 17, 18, 20) and was named ‘loneliness and belonging’. Factor 3 contained four items (SIS: 2, 3, 4, 6) with the exclusion of SIS item 1 as the factor loading was below the cut off (< 0.50) across all factors and this final subscale was named ‘perceived social isolation’.

Post‐hoc reliability analyses showed excellent to good internal consistency for all three factors (Cronbach's alpha *F*1 = 0.95, *F*2 = 0.888 and *F*3 = 0.870). Between factor correlations (correlation coefficients range 0.523–0.649), suggested dependence between factors.

### 3.3 CFA

The three‐factor model proposed by the EFA was submitted to a CFA and assessed using global fit indices on a separate sample (see Table 4 and Supporting Information S1: Figure 1). The CFI value of 0.875 and the TLI (0.856) were below the specified cut offs, suggestive of poor model fit, and the RMSEA (0.110) was larger than the specified cut off again suggesting poor model fit.

**TABLE 4:** CFA Global fit indices for the Stigma Impact Scale (*N* = 353).

	X^2^	df	CFI	TLI	RMSEA
Theoretical model	867.168**	167	0.864	0.845	0.109
Proposed model	784.013**	149	0.875	0.856	0.110

Abbrevaitions: CFI = comparative fit index; df = degrees of freedom; RMSEA = root mean square error of approximation; TLI = Tucker Lewis fit Index; *X* ^*2*^ *=* Chi‐Square goodness of fit.

### 3.4 Endorsement of the SIS

South East Asia (*N* = 29) and Africa (*N* = 8) were not included in the final endorsement table (See Table 5 for WHO Region breakdown and Supporting Information S1: Table 1 for response categories) due to low numbers. In the overall sample, seven items were endorsed by over half the participants. Some of the most commonly endorsed items included item 17 (’‘I feel less competent than I did before my dementia’; 63.52%), item18 (‘I encounter embarrassing situations as a result of my dementia’; 61.55%), item 20 (‘Due to my dementia I sometimes feel useless’; 56.48%). The least commonly endorsed items were item 1 (‘My employer/co‐workers have discriminated against me because of my dementia’; 21.27%), item 5 (‘I feel others are concerned they could catch my dementia through contact like a handshake or eating food I prepare’; 28.03%) and Item 11 (‘I feel a need to keep my dementia a secret’; 32.68%).

**TABLE 5:** Endorsement of the SIS across WHO regions and overall.

	Item wording	European region (*N* = 317)		Region of the Americas (*N* = 241)		West Pacific region (*N* = 110)		Overall sample (*N* = 710)	
		*N*	%	*N*	%	*N*	%	*N*	%
17	I feel less competent than I did before my dementia	127	40.06	202	83.82	91	82.73	451	63.52
18	I encounter embarrassing situations as a result of my dementia	174	54.89	163	67.63	80	72.73	437	61.55
20	Due to my dementia I sometimes feel useless	163	51.42	140	58.09	73	66.36	401	56.48
13	I have a greater need than usual for reassurance that others care about me	140	44.16	135	56.02	72	65.45	379	53.38
2	Some people act as though I am less competent than usual	134	42.27	147	61	61	55.45	377	53.1
14	I feel lonely more often than usual	148	46.69	133	55.19	68	61.82	375	52.82
15	Due to my impairment I have a sense of being unequal in my relationship with others	155	48.9	127	52.7	52	47.27	357	50.28
4	I feel set apart from others who do not have dementia	161	50.79	115	47.72	43	39.09	337	47.46
19	Due to my dementia others seem to feel awkward and tense when they are around me	174	54.89	91	37.76	43	39.09	329	46.34
3	I feel I have been treated with less respect than usual by others	154	48.58	100	41.49	45	40.91	316	44.51
16	I feel I am at least partially to blame for my dementia	178	56.15	125	51.87	48	43.64	296	41.69
12	I feel some friends have rejected me because of my dementia	172	54.26	71	29.46	34	30.91	291	40.99
6	I feel others avoid me because of my dementia	161	50.79	155	64.32	36	32.73	290	40.85
9	I do not feel I can be open with others about my dementia	136	42.9	83	34.44	40	36.36	269	37.89
8	I feel others think I am to blame for my dementia	184	58.04	40	16.6	34	30.91	264	37.18
10	I fear someone telling others about my dementia without my permission	145	45.74	72	29.88	40	36.36	264	37.18
7	Some family members have rejected me because of my dementia	171	53.94	53	21.99	27	24.55	263	37.04
11	I feel a need to keep my dementia a secret	151	47.63	49	20.33	27	24.55	232	32.68
5	I feel others are concerned they could catch my dementia through contact like a handshake or eating food I prepare	169	53.31	16	6.64	11	10	199	28.03
1	My employer/co‐workers have discriminated against me because of my dementia	72	22.71	38	15.77	30	27.27	151	21.27

^a^Endorsement was the proportion of participants who responded either ‘strongly agree’ or ‘agree’.

A graphical representation of the SIS item level endorsement ratings can be found in Supporting Information S1: Figure 2. Participants in the Western Pacific Region (WPR) and the Americas (AMR) endorsed items of the SIS in a similar pattern, the most highly endorsed item was 17 (‘I feel less competent than I did before my dementia’; WPR = 82.73%, AMR = 83.82%) and the least Item 5 (‘I feel others are concerned they could catch my dementia through contact like a handshake or eating food I prepare’ WPR = 10.00%, AMR = 6.64%). In Europe (EUR), the most highly endorsed item was Item 8 (‘I feel others think I am to blame for my dementia’; 58.04%) and the least endorsed (‘My employer/co‐workers have discriminated against me’; 22.71%).

## 4 Discussion

### 4.1 Summary of Findings

The overall aim of this study was to examine the psychometric properties of the SIS in a global sample of people with dementia by examining the reliability, validity and factor structure of the measure as well as levels of endorsement of each item. The SIS and its subscales had adequate to excellent internal consistency. The EFA proposed factor structure did not fully retain any of the original theoretical model subscales but rather reorganised items into new factors. There was a small improvement in the internal consistency observed when transitioning from the theoretical to the EFA‐proposed model primarily due to the removal of one item (SIS1). However, the validity analysis did not support the hypotheses of convergent validity and therefore this psychometric property was not established in this study. Although both the theoretical and EFA‐proposed models were evaluated, neither achieved global fit indices meeting recommended thresholds for good model fit, despite some marginal improvements. All SIS items were endorsed by approximately 20% or more of people with dementia which suggests SIS items reflect relevant and identifiable constructs that resonate with the experience of dementia.

Factor 1 was renamed ‘rejection and secrecy’, it contained nine items which were a combination of the theoretical model subscales of social rejection and internalised shame which adds strength to the argument that perhaps the concepts are intertwined more strongly than originally suggested by the theoretical model hence the analyses of the current study support the creation of a combined rejection and secrecy subscale. Factor 2 comprised six items which were a combination of the theoretical model's entire subscale of social isolation with one addition from the social rejection subscale therefore this factor was named ‘loneliness and belonging’ as the items that referred to internal thoughts and feelings such as a sense of being unequal in relationships or feeling more lonely than usual, all related to an internal sense of loneliness and lack of belonging. Factor 3 comprised four items with the exclusion of SIS 1 as the factor loading was below the cut off. Items within this factor were a combination of the theoretical model subscales of social rejection and internalised shame. Items within this factor focussed on feeling set apart from others, being treated with less respect, perceived avoidance and being perceived as less competent. As the items within this factor all relate to being perceived negatively and therefore set apart or avoided, this factor was named ‘perceived social isolation’.

Overall, the EFA proposed model marginally improved goodness of fit as per the global fit indices however none of the indices met the required cut offs. This suggests that further work on the SIS is necessary in order to understand how to improve the measure. This may involve further changing subscales or looking at whether a bi‐dimensional or unidimensional measure is more appropriate through dropping items or subscales. Future work should consider doing this with people with dementia to ensure the validity of the procedure and relevance of a revised version of the SIS.

### 4.2 Stigma, Wellbeing and Quality of Life in Dementia

It was hypothesised that the more stigma one experiences the poorer one's overall sense of wellbeing would be. However, the validity analysis did not identify a relationship between subjective wellbeing and the SIS. This result aligns with previous findings regarding the relationship between the SIS and self‐esteem including some research which has noted an inverse relationship between self‐esteem and internalised shame specifically [12] and another has found significant negative relationships between all SIS subscales and self‐esteem (Bhatt et al., 2021). Additionally, it is important to consider that wellbeing is culturally sensitive, for example being able to make up one's own mind about things may be a Western representation of positive wellbeing, but in other parts of the world which do not rely on individualistic ideas of decision‐making and autonomy would not be seen as such [29]. There was no significant correlations between stigma impact and quality of life which was not as predicted. It would be plausible to have an inverse relationship emerge between quality of life and stigma however this was not reflected in our data, one reason for this may be due to the diversity within our sample and the notion that quality of life may be a culturally specific phenomenon and can depend on one's expectations of discrimination from others and stance on stigma resistance as a form of empowerment [30].

### 4.3 Endorsement of the SIS

In the overall sample endorsement, items that represented feeling less competent and encountering embarrassing situations aligned to commonly noted stereotypes of dementia which are even more heightened following the divisive and isolating impact of COVID‐19 [11]. Items that were endorsed by > 50% included feeling useless, incompetent, inequality in relationships, loneliness and an increased need for reassurance from others. The latter can be understood through the former list whereby experiencing inequalities in relationships as well as loneliness and being perceived as less competent would understandably result in feeling an increased need for social feedback particularly as meaningful social participation in ones network in dementia is pertinent to manage the condition [31].

## 5 Limitations

As a result of systematically missing data, several cases were excluded from analysis which might have introduced some bias. The missing data may have been due to a technical error or conceptual issue around items 12–20 of the SIS. Although the EFA proposed model requires further improvement, the extent to which items were endorsed speaks volumes to their relevance for people with dementia, perhaps collecting qualitative examples alongside the item responses would have built evidence of face validity. The current study is unable to present findings around the influence of cultural background and stigma experience. The sample in this study was in many ways varied and diverse however due to the small number of participants in some WHO regions or countries, differences between groupings were not analysed as these tests would have been underpowered. Further, the characteristics of the sample were a limitation of this study as the majority of participants were from high‐income countries (81.69%), most of whom had attended higher education (60.6%) and were relatively young (*M* = 64 years of age).

## 6 Conclusion

The SIS appears to be a reliable and well endorsed measure of stigma with people with dementia. Further investigation of factor structure and validity is required and this has implications for future research use. The SIS clearly taps into relevant constructs for people with dementia given the levels of endorsement for each item. This underscores that stigma remains a pervasive issue for many individuals with dementia. The SIS has potential utility beyond research as a guide for clinical interviews or structured interviews to ask about stigma impact may be a fruitful way to understand how health and social care systems can better serve people with dementia. It is beyond the scope of the current study to look into the ethnographic representations of stigma and cultural differences that give rise to and nurture these. Perhaps using the lens of culture, qualitative and quantitative research could deepen our understanding as to how the stigma experience is shaped by various cultural backgrounds.

## Ethics Statement

This research was granted ethical approval by the London School of Economics and Political Science self‐certification process (Reference: CPEC‐LSE‐2019‐SE‐06).

## Conflicts of Interest

The authors declare no conflicts of interest.

## Supporting information

Supporting Information S1

## Data Availability

The data that support the findings of this study are available from Alzheimer's Disease International upon reasonable request.
