# Supplementary material for: An International Validation of the Stigma Impact Scale With People With Dementia
Source: Int J Geriatr Psychiatry. 2025 Jul 3;40(7):e70123. doi: 10.1002/gps.70123 (PMC12231944; doi:10.1002/gps.70123)
Supplement: Supplementary file 1 — Supporting Information S1 [file GPS-40-e70123-s001.docx]

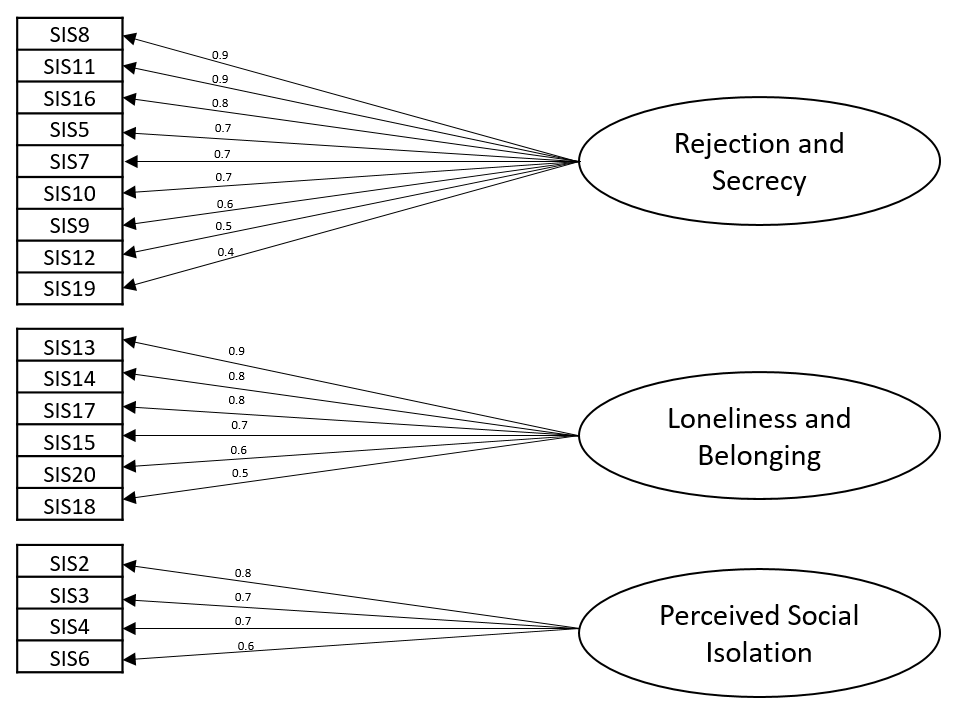


Figure 1. Factor Structure for Stigma Impact Scale based on a confirmatory factor analysis with the three-factor solution

Table 1. Item level responses to the Stigma Impact Scale in the Overall Sample (N=710)

|  | **Item wording** | **Not Applicable** | | **Strongly Agree** | | **Agree** | | **Disagree** | | **Strongly disagree** | |
| --- | --- | --- | --- | --- | --- | --- | --- | --- | --- | --- | --- |
|  |  | N | % | N | % | N | % | N | % | N | % |
| 1 | My employer/co-workers have discriminated against me because of my dementia | 402 | 56.62 | 51 | 7.18 | 100 | 14.08 | 119 | 16.76 | 38 | 5.35 |
| 2 | Some people act as though I am less competent than usual | 113 | 15.92 | 74 | 10.42 | 303 | 42.68 | 178 | 25.07 | 42 | 5.92 |
| 3 | I feel I have been treated with less respect than usual by others | 101 | 14.23 | 74 | 10.42 | 242 | 34.08 | 246 | 34.65 | 47 | 6.62 |
| 4 | I feel set apart from others who do not have dementia | 96 | 13.52 | 78 | 10.99 | 259 | 36.48 | 234 | 32.96 | 43 | 6.06 |
| 5 | I feel others are concerned they could catch my dementia through contact like a handshake or eating food I prepare | 94 | 13.24 | 115 | 16.20 | 84 | 11.83 | 189 | 26.62 | 228 | 32.11 |
| 6 | I feel others avoid me because of my dementia | 93 | 13.10 | 81 | 11.41 | 209 | 29.44 | 232 | 32.68 | 95 | 13.38 |
| 7 | Some family members have rejected me because of my dementia | 109 | 15.35 | 103 | 14.51 | 160 | 22.54 | 195 | 27.46 | 143 | 20.14 |
| 8 | I feel others think I am to blame for my dementia | 97 | 13.66 | 94 | 13.24 | 170 | 23.94 | 188 | 26.48 | 161 | 22.68 |
| 9 | I do not feel I can be open with others about my dementia | 79 | 11.13 | 62 | 8.73 | 207 | 29.15 | 251 | 35.35 | 111 | 15.63 |
| 10 | I fear someone telling others about my dementia without my permission | 92 | 12.96 | 74 | 10.42 | 190 | 26.76 | 246 | 34.65 | 108 | 15.21 |
| 11 | I feel a need to keep my dementia a secret | 87 | 12.25 | 79 | 11.13 | 153 | 21.55 | 242 | 34.08 | 149 | 20.99 |
| 12 | I feel some friends have rejected me because of my dementia | 103 | 14.51 | 101 | 14.23 | 190 | 26.76 | 229 | 32.25 | 87 | 12.25 |
| 13 | I have a greater need than usual for reassurance that others care about me | 80 | 11.27 | 78 | 10.99 | 301 | 42.39 | 205 | 28.87 | 46 | 6.48 |
| 14 | I feel lonely more often than usual | 78 | 10.99 | 92 | 12.96 | 283 | 39.86 | 207 | 29.15 | 50 | 7.04 |
| 15 | Due to my impairment I have a sense of being unequal in my relationship with others | 80 | 11.27 | 75 | 10.56 | 282 | 39.72 | 221 | 31.13 | 52 | 7.32 |
| 16 | I feel I am at least partially to blame for my dementia | 79 | 11.13 | 99 | 13.94 | 197 | 27.75 | 185 | 26.06 | 150 | 21.13 |
| 17 | I feel less competent than I did before my dementia | 61 | 8.59 | 137 | 19.30 | 314 | 44.23 | 156 | 21.97 | 42 | 5.92 |
| 18 | I encounter embarrassing situations as a result of my dementia | 81 | 11.41 | 104 | 14.65 | 333 | 46.90 | 160 | 22.54 | 32 | 4.51 |
| 19 | Due to my dementia others seem to feel awkward and tense when they are around me | 86 | 12.11 | 63 | 8.87 | 266 | 37.46 | 245 | 34.51 | 50 | 7.04 |
| 20 | Due to my dementia I sometimes feel useless | 75 | 10.56 | 87 | 12.25 | 314 | 44.23 | 179 | 25.21 | 55 | 7.75 |

Figure 2. Endorsement of SIS items across four WHO Region

| **Table 2**  Participant demographics variables– full table | | | | | | |
| --- | --- | --- | --- | --- | --- | --- |
| Variable | **N(%) or Mean(SD)** | | | Variable | **N(%)** | |
| Sex | Male | | 277(39.00) | Country | Argentina | 2(0.28%) |
|  | Female | | 433(61.00) |  | Australia | 30(4.23%) |
| Age | N=710,  Range: 24-92 | | 64.81(11.71) |  | Belgium | 12(1.69%) |
| Employment status | Full time paid employment | | 101(14.20) |  | Brazil | 36(5.07%) |
|  | Part time paid employment | | 26(3.70) |  | Canada | 27(3.80%) |
|  | Self-employed | | 50(7.00) |  | China | 11(1.55%) |
|  | Unpaid/voluntary work | | 55(7.70) |  | Colombia | 4(0.56%) |
|  | Unpaid carer | | 16(2.30) |  | Costa Rica | 3(0.42%) |
|  | Retired | | 391(55.10) |  | Croatia | 2(0.28%) |
|  | Student | | 4(0.60) |  | Dominican Republic | 1(0.14%) |
|  | Illness/sick-leave | | 43(6.10) |  |  |  |
|  | Looking for/other, unemployed | | 54(7.60) |  | France | 2(0.28%) |
|  |  |  |  |  | Germany | 3(0.42%) |
| Education | Less than primary/elementary school | | 5(0. 70) |  | Greece | 7(0.99%) |
|  |  |  |  |  | Iceland | 1(0.14%) |
|  | Primary/elementary school | | 22(3.10) |  | India | 14(1.97%) |
|  | Secondary school/ High school (or equivalent) | | 163(23.00) |  | Indonesia | 9(1.27%) |
|  | Vocational training or apprenticeship | | 90(12.70) |  | Iran | 2(0.28%) |
|  |  |  |  |  | Ireland | 1(0.14%) |
|  |  |  |  |  | Italy | 59(8.31%) |
|  | College/pre-university/university | | 257(36.20) |  | Japan | 9(%) |
|  |  |  |  |  | Kenya | 1(0.14%) |
|  | Post graduate degree completed | | 173(24.40) |  | Lebanon | 1(0.14%) |
| Area of residence | Urban | | 332(45.40) |  | Malaysia | 7(0.99%) |
|  | Suburban | | 162(22.80) |  | Mauritius | 1(0.14%) |
|  | Semi-rural | | 155(21.80) |  | Mexico | 8(1.13%) |
|  | Rural | | 59(8.30) |  | Netherlands | 181(25.49%) |
| Stigma Impact Scale | N= 710 | Range: 78.00 | 42.35(16.38) |  | New Zealand | 15(2.11%) |
|  |  |  |  |  | Norway | 1(0.14%) |
|  |  |  |  |  | Philippines | 3(0.42%) |
|  |  |  |  |  | Portugal | 2(0.28%) |
| WEMWBS total | N= 681 | Range: 1.70 | 44.40(11.28) |  | Puerto Rico | 1(0.14%) |
| DEMQoL total | N= 596 | Range:2.79 | 1.99(0.30) |  | Qatar | 2(0.28%) |
| WEMWBS categorical | Higher mental wellbeing ≥42 | | 408(57.46%) |  | Russia | 3(0.42%) |
|  | Lower mental wellbeing (0-41) | | 266(37.46%) |  | Singapore | 1(0.14%) |
| DQoL categorical | Higher QoL (> median 2.25) | | 103(14.51%) |  | Slovenia | 3(0.42%) |
|  | Lower QoL (< median 2.25) | | 493(69.43%) |  | South Africa | 6(0.85%) |
| WHO Region | African Region | | 8(1.13%) |  | Spain | 2(0.28%) |
|  | Eastern Mediterranean Region | | 5(0.70%) |  | Taiwan | 20(2.82%) |
|  | European Region | | 317(44.65%) |  | Thailand | 6(0.85%) |
|  | Region of the Americas | | 241(33.94%) |  | United Kingdom | 38(5.35%) |
|  | South-East Asia Region | | 29(4.08%) |  | United States | 159(22.39%) |
|  | Western Pacific Region | | 110(15.49%) |  | Vietnam | 14(1.97%) |
| World bank income categories | High-income economies | | 580(81.69%) |  |  | |
|  | Upper-middle economies | | 89(12.54%) |  |  |  |
|  | Lower-middle economies | | 41(5.77%) |  |  |  |
